# Nucleation of Surfactant–Alkane Mixed Solid Monolayer and Bilayer Domains at the Air–Water Interface

**DOI:** 10.3390/ma15020485

**Published:** 2022-01-09

**Authors:** Hiroki Matsubara, Rikako Mori, Eisuke Ohtomi

**Affiliations:** 1 Department of Chemistry, Faculty of Science, Kyushu University, Motooka 744, Nishi-ku, Fukuoka 819-0395, Japan; cosmos.r.mori@gmail.com (R.M.); Eisuke.Ohtomi@wdc.com (E.O.); 2 Graduate School of Advanced Science and Engineering, Hiroshima University, Kagamiyama 1-3-1, Higashi, Hiroshima 739-8526, Japan

**Keywords:** surfactant, alkane, wetting, surface freezing, phase transitions, line tension

## Abstract

We investigated the wetting transitions of tetradecane and hexadecane droplets in dodecyltrimethylammonium bromide (C12TAB), tetradecyltrimethylammonium bromide (C14TAB), and hexadecyltrimethylammonium bromide (C16TAB) aqueous solutions. By varying the surfactant concentration, the formation of mixed monolayers of a surfactant and an alkane was observed at the air–water interface. Depending on the combination of surfactant and alkane, these wetting monolayers underwent another thermal phase transition upon cooling either to a frozen mixed monolayer (S1) or a bilayer structure composed of a solid monolayer of a pure alkane rested on a liquid-like mixed monolayer (S2). Based on the phase diagrams determined by phase modulation ellipsometry, the difference in the morphology of the nucleated S1 and S2 phase domains was also investigated using Brewster angle microscopy. Domains of the S1 phase were relatively small and highly branched, whereas those of the S2 phase were large and circular. The difference in domain morphology was explained by the competition of the domain line tension and electrostatic dipole interactions between surfactant molecules in the domains.

## 1. Introduction

It is well known that the 2D analogue of surface tension, called line tension, arises at the boundary between two surface phases. Line tension has been discussed in its relation to the lipid raft formation in cellular membranes [1,2,3,4]. One of the main origins of line tension is a difference in height between coexisting ordered and disordered lipid domains. The ordered phase (lipid raft) is rich in sphingolipids and cholesterol, and compared to the surrounding disordered phase, the chains of lipids are more saturated. This lipid composition allows the formation of highly packed ordered chains, and, as a result, the ordered domains become thicker than the surrounding disordered phase. Microscopic phase separation has been explored extensively because it is related to some important biological functions such as molecular recognition, and ion transfer through cellular membranes [5,6,7]. Therefore, understanding the physicochemical origin of domain formation is a topical issue in membrane science.

On the other hand, monolayers of surfactant molecules adsorbed at the air–water interface have 2D gas (G), liquid (L), and solid (S) phases, similar to 3D materials, distinguished by the abrupt change in surface density observed when the surfactant concentration or temperature is varied. Among these surface phases, only sparingly water-soluble amphiphiles, such as long-chain alcohols, show a 2D solid phase. This is because ionic surfactants and nonionic surfactants with large hydration shells generate in-plane repulsions between their hydrophilic groups, therefore hindering the formation of highly condensed films.

However, as we showed previously [8,9,10], surfactant–alkane mixed adsorbed films underwent a freezing transition upon cooling when alkane molecules penetrated the surfactant adsorbed film during the wetting transition (pseudo-partial wetting). Interestingly, the low-temperature phase of the surfactant–alkane mixed monolayer has two distinctive structures depending on the combination of surfactant and alkane: a frozen mixed monolayer (S1), and a bilayer structure where the upper layer is a solid alkane monolayer and the lower layer is a disordered mixed monolayer (S2).

Deutsch et al. [11] studied the wetting transitions of *n*-alkanes of various chain lengths in aqueous hexadecyltrimethylammonium bromide (C16TAB) and showed that an S2 film formed for octadecane and longer alkanes, while for alkanes with chain lengths similar to or shorter than C16TAB, S1 film formation dominated. They also applied X-ray reflectometry to determine the molecular-level structures of these surface frozen films.

The type of surface frozen phase can also be determined by ellipsometry because the coefficient of ellipticity obtained for the S1 and S2 phases is largely different. Using this technique, we created a 2D phase diagram for the wetting films of tetradecane (C14) in tetradecyltrimethylammonium bromide (C14TAB) aqueous solution and hexadecane (C16) in dodecyltrimethylammonium bromide (C12TAB) aqueous solution as a function of the temperature and surfactant concentration in the aqueous phase [8,9]. The S1 and S2 phases were exhibited for the C14TAB-C14 and C12TAB-C16 systems, respectively.

In this paper, we performed ellipsometric measurements to construct 2D phase diagrams for six combinations of alkanes (C14 and C16) and surfactants (C12-, C14-, and C16TAB). We made these selections because surface freezing for shorter-chain alkanes is only observed in a supercooled state, and there is a solubility limit for longer-chain surfactants. In addition, based on the obtained 2D phase diagrams, the morphology of the nucleated S1 and S2 domains in the liquid-like mixed monolayer (L) was investigated by using Brewster angle microscopy (BAM). The size and shape of domains were determined by line tension and electrostatic interactions between dipoles on cationic surfactant head groups. The former favors a circular domain with minimum line energy, whereas the latter favor smaller and elongated domains [12,13,14]. Compared to lipid membranes, the extent of height mismatch between the L, S1, and S2 phases can be readily controlled for surfactant–alkane mixed adsorbed films, and the difference in electric charge density (i.e., surface density of cationic surfactants) can also be obtained by the analysis of surface tension data through the Gibbs adsorption isotherm. Hence, the present experiments propose a qualitative physicochemical understanding of the nucleation of ordered domains from the comparison between domain morphologies.

## 2. Materials and Methods

Dodecyltrimethylammonium bromide (C12TAB, Tokyo Chemical Industry, Tokyo, Japan, >98%), tetradecyltrimethylammonium bromide (C14TAB, Wako Chemicals, >99%), and hexadecyltrimethylammonium bromide (C16TAB, Nacalai Tesque, Kyoto, Japan, >99%) were purified through recrystallization from a mixture of acetone and ethanol. Oil-soluble impurities were extracted from their solid powders using hexane. The purities of all the surfactants were confirmed by the absence of a minimum on the surface tension vs. concentration curves around their critical micelle concentrations (CMCs). All samples were prepared with Milli-Q water. *n*-Tetradecane (C14, Nacalai tesque, Kyoto, Japan, >98%) and *n*-hexadecane (C16, Nacalai tesque, Kyoto, Japan, >98%) were distilled under reduced pressure before use.

Ellipsometric measurements were performed using a Picometer Ellipsometer (Beaglehole Instruments, Wellington, New Zealand) equipped with a HeNe laser (λ = 632.8 nm). The surfactant solution contained in a 5 cm-diameter glass dish was thermostated by water circulation in a copper jacket. Then, a small amount of the alkane was spread on the solution surface with a spreading agent (chloroform). The temperature of the surrounding air was controlled separately using another water circulation system. The coefficient of ellipticity $\bar{\rho }$ defined as the imaginary part of $r_{p}/r_{s}$ at a Brewster angle of ~53° was measured. Here, $r_{p}$ and $r_{s}$ are the complex Fresnel reflection coefficients for p- and s-polarized lights, respectively.

For Brewster angle microscopy (BAM: Nanofilm microscope, Accurion, Göttingen, Germany), a Langmuir trough was first filled with the surfactant solution. After the liquid alkane was spread on the solution surface, in keeping with the ellipsometric measurements, the temperature of the Langmuir trough was continuously decreased to capture the subsequent images of surface freezing processes. The temperature of the solution was monitored using a Pt resistance thermometer during the BAM observation, with a precision of 0.1 K.

We also performed surface tension measurements for the C14TAB-C14, C16TAB-C14, and C16TAB-C16 systems to determine the surface density of surfactants in the L and S1 phases. To measure the surface tension of surfactant–alkane mixed adsorbed films, pendant drop measurement was conducted in a closed glass cell saturated with alkane vapor. The experimental error in the interfacial measurements was <0.1 mN m^−1^.

## 3. Results

### 3.1. Ellipsometry

**Figure 1:**
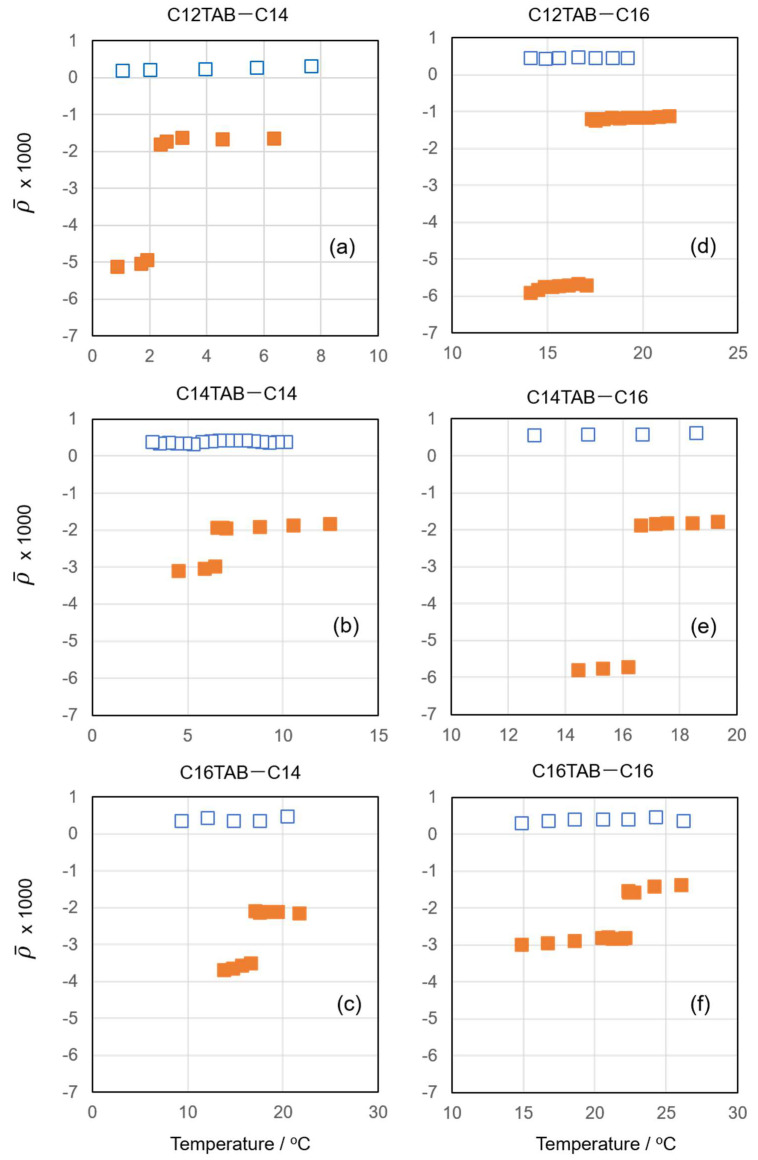
Coefficient of ellipticity vs. temperature plots for (**a**) C12TAB + C14 at surfactant concentrations *m* = 0.20 (blue open squares) and 4.00 (orange filled squares) mmol kg^−1^, (**b**) C14TAB + C14 at surfactant concentrations *m* = 0.05 (□) and 1.50 (■) mmol kg^−1^, (**c**) C16TAB + C14 at *m* = 0.02 (□) and 0.41 (■) mmol kg^−1^, (**d**) C12TAB + C16 at *m* = 0.20 (□) and 4.00 (■) mmol kg^−1^, (**e**) C14TAB + C16 at *m* = 0.05 (□) and 3.50 (■) mmol kg^−1^, and (**f**) C16TAB + C16 at *m* = 0.02 (□) and 0.40 (■) mmol kg^−1^.

Figure 1 shows the coefficient of ellipticity $\bar{\rho }$ measured as a function of temperature for different combinations of surfactant and alkane. For each experimental system, ellipticity data taken at two different concentrations were selected. The $\bar{\rho }$ values obtained at low concentrations were positive in all the experimental systems. The positive contribution to $\bar{\rho }$ observed here comes from the thermal roughness of the air–water interface [15]. For the surface of pure water, $\bar{\rho }$ is determined by this thermal roughness, and at 298.15 K, it is about $\bar{\rho }\sim 0.4\times 10^{-3}$. This value is very close to the $\bar{\rho }$ values obtained at low concentrations. Therefore, it can be concluded that at these concentrations, only a negligible amount of the surfactant and oil is present at the air–water interface, i.e., these adsorbed films are in the gas state.

When we measured $\bar{\rho }$ at high concentrations, however, it became negative and abruptly decreased at a certain temperature upon cooling. The high temperature phase is assumed to be a pseudo-partial wetting film in the L state, and the low temperature phase might be either an S1 or S2 surface frozen film. In the L state, the surfactant–alkane mixed adsorbed film can be considered as an isotropic hydrocarbon monolayer of thickness *d* and permittivity *ε*. In this case, $\bar{\rho }$ can be expressed by the Drude equation [16,17,18]:

(1) $${\bar{\rho }}_{L}=\frac{\pi }{\lambda }\sqrt{\frac{\varepsilon_{1}+\varepsilon_{2}}{\varepsilon_{1}-\varepsilon_{2}}}\frac{\left(\varepsilon -\varepsilon_{1}\right)\left(\varepsilon -\varepsilon_{2}\right)}{\varepsilon }d$$

where λ is the wavelength of light, and $\varepsilon_{1}$ and $\varepsilon_{2}$ are the relative permittivities of the air and water, respectively. For example, when we adopt the permittivity of liquid hexadecane (ε= 2.05) [19] and *d* = 1.38 nm determined previously by X-ray reflectometry [10], ${\bar{\rho }}_{L}$ is estimated as −1.5 × 10^−3^ for the C16TAB + C16 system. In order to calculate $\bar{\rho }$ more precisely, we need to take account of the hydrophilic group layer and counterion contributions. However, as ${\bar{\rho }}_{L}$ is the main contribution of $\bar{\rho }$, the reasonable coincidence between the calculated ${\bar{\rho }}_{L}$ and measured $\bar{\rho }$ supports that the high-temperature phase of the surfactant–alkane mixed monolayer is in the liquid state.

On the other hand, the frozen monolayer (S1) is optically anisotropic, and the Drude equation can be rewritten as [20]:

(2) $${\bar{\rho }}_{S}=\frac{\pi }{\lambda }\sqrt{\frac{\varepsilon_{1}+\varepsilon_{2}}{\varepsilon_{1}-\varepsilon_{2}}}\left\{\frac{\left(\varepsilon_{e}-\varepsilon_{1}\right)\left(\varepsilon_{e}-\varepsilon_{2}\right)}{\varepsilon_{e}}+\left(\varepsilon_{o}-\varepsilon_{e}\right)\right\}d$$

where $\varepsilon_{e}$ and $\varepsilon_{o}$ are the permittivities of the mixed adsorbed film perpendicular and parallel to the interface, respectively. Using $\varepsilon_{e}$ = 2.33 and $\varepsilon_{o}$ = 2.21 determined for a hydrocarbon crystal [20,21] and *d* = 2.17 nm [10], Equation (3) gives ${\bar{\rho }}_{S}\sim$−3.9 × 10^−3^, which is in good agreement with the $\bar{\rho }$ values in the low-temperature regions in Figure 1b,c,f. Considering that the S2 film is composed of a frozen alkane monolayer on the liquid-like monolayer, its ellipticity can be approximated by the sum of ${\bar{\rho }}_{L}$ and ${\bar{\rho }}_{S}$ (~−5.4 × 10^−3^). In actuality, the $\bar{\rho }$ values observed in Figure 1a,d,e in the low-temperature region coincide well with this estimation.

**Figure 2:**
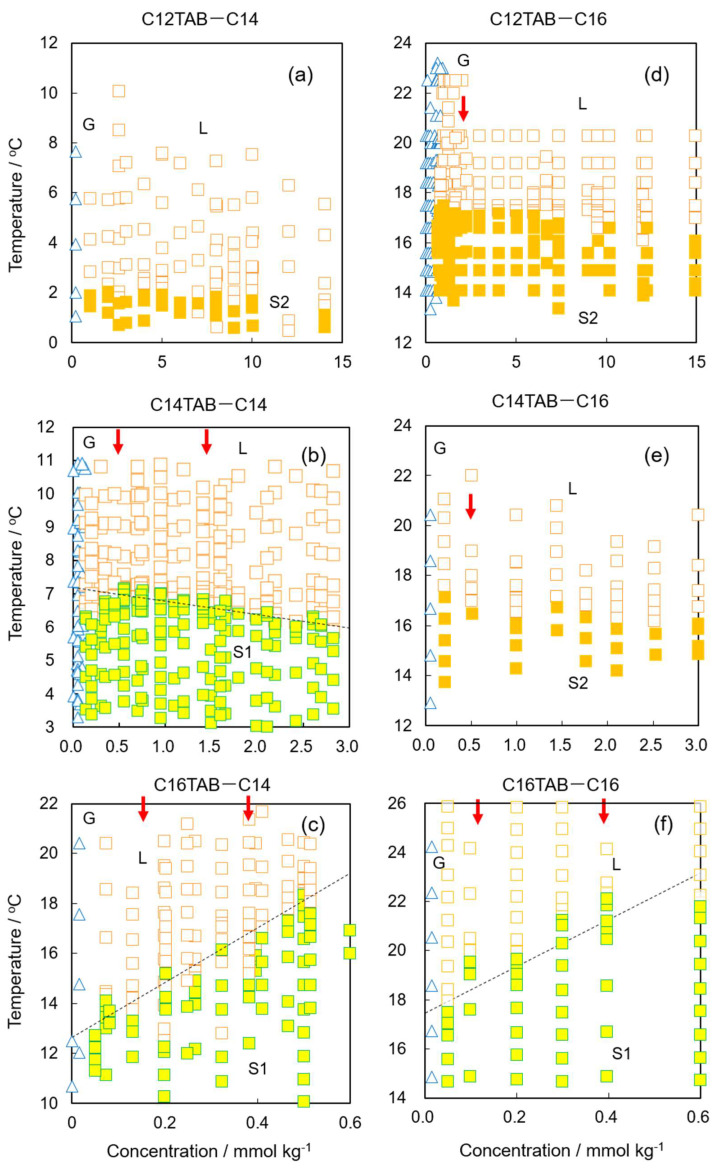
Surface phase diagrams for (**a**) C12TAB + C14, (**b**) C14TAB + C14, (**c**) C16TAB + C14, (**d**) C12TAB + C16, (**e**) C14TAB + C16, and (**f**) C16TAB + C16 systems. Blue open triangles and orange open squares are the G and L states, respectively. Orange and yellow filled squares are the S2 and S1 states, respectively. Red arrows in each diagram show experimental conditions where Brewster angle microscopy was applied.

We repeated the same experiments at various surfactant concentrations and summarized the obtained data as temperature–surfactant concentration diagrams (Figure 2). Comparing Figure 2a–c and Figure 2e,f, it was found that the structure of the surface frozen film changed from S2 to S1 as the surfactant chain length increased with the same alkane. In order to induce S2 film formation, the alkane chain length must be at least two methylene units longer than the hydrophobic chains of the surfactant, i.e., an S2 film can be formed with C12TAB when using tetradecane as an oil, whereas it can also be formed with C14TAB when using hexadecane. In such a situation, the surfactant and alkane molecules cannot generate enough van der Waals attraction to create an S1 film due to the chain mismatch, and therefore the frozen monolayer formation of alkane molecules (S2 film formation) dominates the phase behavior. The transition temperature does not strongly depend on the surfactant concentration in the aqueous phase, as seen in Figure 2a,d,e. However, the boundary between L and S1 states slightly decreased with increasing surfactant concentration with C14TAB (Figure 2b) and increased more clearly with C16TAB (Figure 2c,f).

### 3.2. Brewster Angle Microscopy

**Figure 3:**
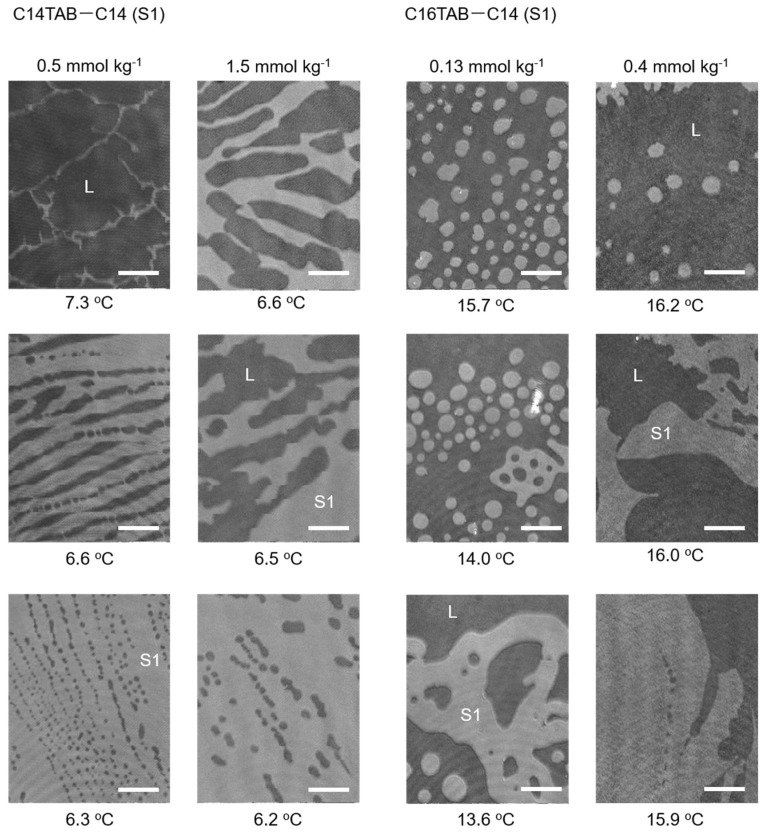
BAM images of nucleated S1 domains for the C14TAB−C14 system (left two columns) and those for the C16TAB−C14 system (right two columns). Images for the C14TAB−C14 system at 0.5 mmol kg^−1^ were adapted with permission from Ref. [22] (H. Matsubara et. al, *J. Phys. Chem. Lett*. **2013**, *4*, 844–848. Copyright 2013 American Chemical Society).

**Figure 4:**
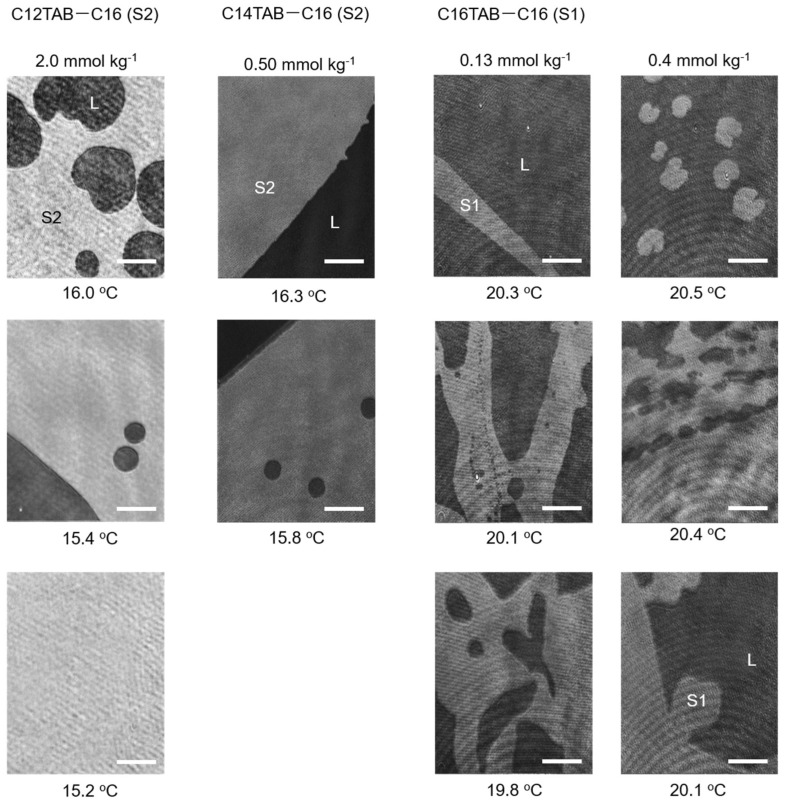
BAM images of nucleated S2 domains for the C12TAB−C16 system (left column) and those for the C14TAB−C16 system (second column from left). The right two columns show BAM images of S1 domains taken for the C16TAB−C16 system. Images for the C14TAB−C16 system were reproduced from Ref. [22] with permission (H. Matsubara et al., *J. Phys. Chem. Lett*. **2013**, *4*, 844–848. Copyright 2013 American Chemical Society).

The results of the BAM measurements are summarized in Figure 3 and Figure 4. For the C12TAB-C14 system, the transition temperature between the L and S2 phases was around 2 °C; therefore, performing BAM observation under precise temperature control was difficult. For the C12TAB-C16 and C14TAB-C16 systems, large circular S2 phase domains (brighter region in the BAM image) appeared around 16 °C, which is slightly below their surface phase transition temperature (see Figure 2d,e). Then, the S2 phase domains merged and covered the whole field of the microscope within 1 to 2 °C below the surface phase transition temperature. The structure of the S2 phase domains did not depend strongly on the concentration of surfactant in the aqueous phase; hence, the BAM images taken at a single surfactant concertation were depicted for these systems. Surfactant concentrations at which the BAM measurements were performed are described at the top of the BAM images, and by red arrows in the surface phase diagrams shown in Figure 2.

Contrary to S2 domain formation, the structures observed for the S1 domain were more complicated. In the C14TAB-C14 system, we first observed a network of thin S1 domains that evolved into a thicker stripe with decreasing temperature. When the C14TAB concentration was increased, the width of the stripe domain increased. The boundaries between the S1 domain and its surrounding L phase also had spike shapes, which were not observed in other S1 domains. For example, in the C16TAB-C14 system, the S1 domains first appeared as a deformed circular shape. However, the size of the circular domains was much smaller than that of the S2 domain, and, as a result, the number of nucleated domains was also greater than that of the S2 domains. These domains then merged into a larger domain, causing the perimeter to curve in an irregular manner and protrude at some points. Similar behavior was also observed in the C16TAB-C16 system, although the size of the S1 domain looked slightly larger and the curvature of the phase boundary seemed to be less in comparison to the C16TAB-C14 system. Hence, it may be stated that the nucleation behavior in this system is intermediate between the C14TAB-C14 and C16TAB-C14 systems from the viewpoint of their morphologies.

## 4. Discussion

As mentioned in the Introduction, the morphology of solid domains can be determined by the competition of the domain line tension *τ* and the electric repulsions of hydrophilic groups in the solid domain. The most simple expression for domain line tension is given by

(3) τ=γΔl

where *γ* is the surface tension of hydrocarbon chains exposed to the air at the domain boundary (~25 mJ m^−2^), and Δ*l* is the height mismatch between the solid domain and the surrounding L phase. McConnell et al. [23,24] proposed a theoretical model for the interaction energy of an isolated circular domain of radius *R*, where molecular dipoles are oriented perpendicular to the air–water interface, as

(4) $$E=2\pi R\left[\tau -\frac{m^{2}}{4\pi \varepsilon_{2}\varepsilon_{0}}\ln\frac{4R}{e^{2}\delta }\right]$$

where *m* is the dipole moment density difference between the domain and its surroundings, $\varepsilon_{0}$ and $\varepsilon_{2}$ are the permittivities of the vacuum and water, and δ is the cut-off distance between dipoles in the solid domain.

**Figure 5:**
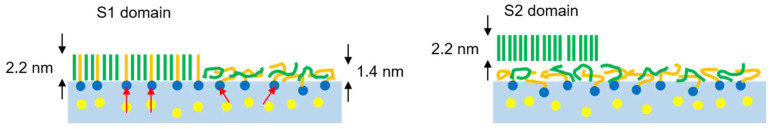
Schematic illustrations for the S1 domain of the C16TAB-C16 system (**left**) and for the S2 domain of the C14TAB-C16 system (**right**). Red arrows in the aqueous phase are dipoles on the hydrophilic groups. Surfactant hydrophobic chains and alkane molecules are, respectively, colored by orange and green to illustrate the difference visibly.

For S2 phase domains, Δ*l* is roughly equal to the all-trans chain length of the alkanes examined (see Figure 5, right). Hence, the line tension of the C14 (Δ*l*~1.9 nm) and C16 (~2.2 nm) S2 domains on the 2D liquid film can be estimated as ~4.8 × 10^−11^ and ~5.5 × 10^−11^ J/m, respectively. On the other hand, the opposing electric repulsion term is practically negligible because the S2 domain is composed of an island of frozen alkane molecules floating on the 2D liquid. Therefore, line tension dominates the phase behavior, and the domains become large and circular in order to minimize the energy penalty of the perimeter, as demonstrated in the BAM images taken for the C12TAB-C16 and C14TAB-C16 systems.

For the S1 phase domains, however, the line tension contribution is significantly decreased. In the C16TAB-C16 system, the thickness of S1 and L films determined by X-ray reflectometry [10] was 2.2 and 1.4 nm, respectively. Using these values, Δ*l* can be estimated as 0.8 nm, and the resultant τ is ~2.0 × 10^−11^ J/m, which is about 1/2~1/3 compared to that estimated for the S2 phase domains. In addition, this line tension contribution is further diminished by molecular dipole contributions. In the L phase, the trimethylammonium head groups and counterions arrange themselves to minimize unfavorable electrostatic interactions. However, such a rearrangement is difficult in the S1 phase because the chain packing between hydrophobic chains and alkane molecules leads to a coplanar arrangement for the head groups. As a result, all dipoles created between the TMA^+^ ions and the adjacent Br^−^ ions in the Stern layer are lined up, leading to a large dipole density difference between the S1 domains and the surrounding L phase (see Figure 5, left).

**Figure 6:**
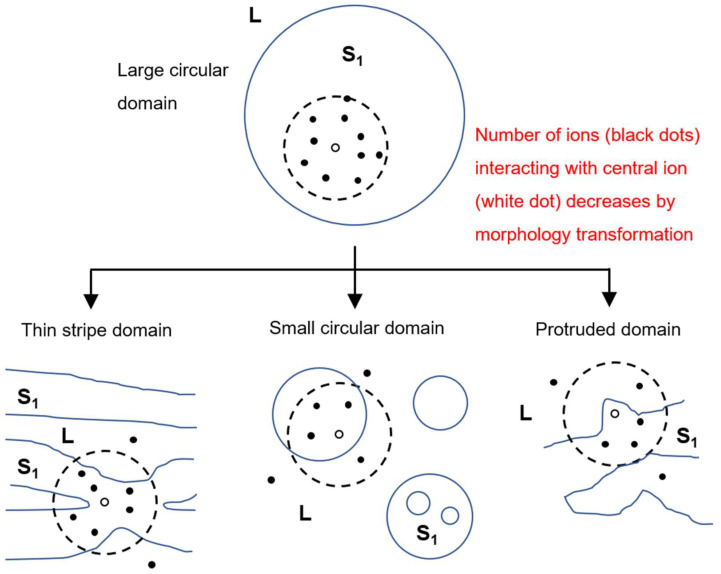
Schematic diagrams of dipole repulsion exerted on an arbitrary surfactant ion (open circle) from other surfactant ions (filled circles) in the interaction range (dotted circle). Transformation from a large circular domain into thin-striped, small, circular or protruded domains reduces the number of dipoles in the interaction range.

We had previously interpreted the domain morphology observed for C14TAB-C14 (S1) and C14TAB-C16 (S2) systems according to the abovementioned concept [22]. In this study, we investigated the change in the morphology of S1 domains more closely by comparing the BAM images taken for the C14TAB-C14, C16TAB-C14, and C16TAB-C16 systems. Table 1 is a summary of the surface densities of surfactants at concentrations where BAM observation was performed (see SI for the original surface tension data). Here, the chosen surfactant concentrations were selected as their surface densities—both in the L and S1 states—were roughly equal between the three systems, i.e., the surface densities at the lower concentrations were ~2.0 μmol m^−2^, and those at higher concentrations were ~3.0 μmol m^−2^. Using these selections, it can be assumed that the electric repulsion contribution to the domain morphology is roughly equal between the three experimental systems. Therefore, the difference in domain morphology observed here is mainly attributed to the difference in line tension effects between the three systems. From this point of view, the thin S1 domains with sharp spikes observed in the C14TAB-C14 system can be explained by its low line tension due to its short hydrocarbon chains (C14 chains). In such a case, the electric repulsion exerted on an arbitrary surfactant ion (open circle in Figure 6) decreased when a larger circular domain deformed into a thread-like or stripe-shaped domain. This was also true for spikes on the domain perimeter, and the formation of smaller circular domains, as shown in Figure 6. The S1 domains observed here seemed to reduce their electric dipole repulsions through any of these transformations or combinations of them during nucleation.

Considering the average chain length of the surfactant and alkane contained in the S1 domain, the magnitude of the line tension seemed to increase in the order of C14TAB + C14 < C16TAB + C14 < C16TAB + C16. For example, the S1 domains that first appeared in the C16TAB-C14 system were small circular ones, which then merged into a larger irregular shaped domain as the temperature decreased. This indicates that the line tension of S1 domains observed in the C16TAB + C14 system was higher in comparison to that of those in the C14TAB-C14 system. This increment in line tension seems to suppress the transformation of the domain morphology into striped domains. However, going by how the size of circular domains was much smaller than that observed for S2 domains, and that the domains eventually grew into an irregularly shaped network, it can be concluded that electric dipole repulsion still dominates the nucleation process on the whole. In the C16TAB-C16 system, the line tension of which is expected to be of the largest value among the three systems, a relatively large domain is formed immediately after the phase transition, although the domain shape is still irregular with flexible curvatures.

When the surfactant concentration is increased in the same system, the growth rate of the domain seems to be faster, and large domains can be more easily found under the microscope. As shown in Table 1, the surfactant ions contained in the S1 domain increased at higher concentrations. Intuitively, the increase in surface density seems to promote domain deformations due to stronger dipole repulsions as compared to the low-surfactant concentration experiments. However, the domain size increased contrary to this simple expectation. Some researchers [2,4,25] proposed an elastic deformation model for domain line tension, in which the domain boundary stores mechanical energy accompanied by stretching or shrinking of hydrocarbon chains to reduce their exposure to the surrounding phase. It is reasonable to assume that an increase in the surfactant content in the S1 domain promotes the upright orientation of hydrocarbon chains. Therefore, the elastic deformation at the domain boundary is probably more suppressed as the surfactant concentration increases, and, as a result, there is an increase in domain line tension as compared to the case where hydrocarbon chains are more freely deformed. In addition, it should also be considered that the high surface density of the surfactant generally increases the number of nucleated S1 domains. Therefore, another possibility of the existence of a larger domain at high-concentration regimes may be explained by the merging of many initially nucleated domains into irregularly patterned domains. This effect should be especially pronounced for S1 domains contained in C16TAB owing to its stronger tendency to stabilize S1 films, which is more pronounced with an increasing concentration, as seen through the rise in the L–S1 phase transition temperature in Figure 2c,f.

In this paper, we demonstrated that the morphology of nucleated solid domains of surfactant–alkane mixed films is determined by the competition between domain line tension and electric dipole repulsions between surfactant head groups. A small change in the hydrocarbon chain length significantly changed the shape of the S1 domain from small, circular, thin-striped to protruded domains, indicating that the dipole repulsion term dominated the morphology of the domains in the experimental conditions examined here. For ordered domains of lipids, it is believed that the size of the domains is in the range of several tens of nanometers. In order to create such a small domain, it is necessary to reduce the line tension term by a considerable extent. According to this line, similar to surfactants in 3D systems, it has been proposed that some type of molecule acts as a line-active agent (linactant) which stabilizes 2D domains [26,27]. Studying the effect of linactants on the domain morphology of surfactant–alkane mixed monolayers is therefore an interesting future extension of the present work.

## Figures and Tables

**Table 1:** Bulk concentration and surface density of surfactants at the experimental points applied to BAM experiments.

C14TAB-C14	C16TAB-C14	C16TAB-C16
0.5 mmol kg^−1^	0.13 mmol kg^−1^	0.13 mmol kg^−1^
(L) 2.1 μmol m^−2^	(L) 1.8 μmol m^−2^	(L) 2.0 μmol m^−2^
(S1) 2.2 μmol m^−2^	(S1) 2.4 μmol m^−2^	(S1) 2.3 μmol m^−2^
1.5 mmol kg^−1^	0.4 mmol kg^−1^	0.4 mmol kg^−1^
(L) 3.0 μmol m^−2^	(L) 2.9 μmol m^−2^	(L) 3.0 μmol m^−2^
(S1) 2.9 μmol m^−2^	(S1) 2.9 μmol m^−2^	(S1) 3.0 μmol m^−2^

Surface densities of surfactants were obtained from surface tension measurements. The original surface tension data are available in the Supplementary Materials Figures S1 and S2.

## Data Availability

The data presented in this paper are available on request from the corresponding author.

## Supplementary Materials

The following are available online at https://www.mdpi.com/article/10.3390/ma15020485/s1, Figure S1. Surface tension (open circle) and surface density (filled circle) vs. C16TAB concentration curves in the presence of C14 at (1) 25.0 °C and (2) 10.0 °C, Figure S2. Surface tension (open circle) and surface density (filled circle) vs. C16TAB concentration curves in the presence of C16 at (1) 25.0 °C and (2) 19.0 °C.
