# Supplementary material for: Nucleation of Surfactant–Alkane Mixed Solid Monolayer and Bilayer Domains at the Air–Water Interface
Source: Materials (Basel). 2022 Jan 9;15(2):485. doi: 10.3390/ma15020485 (PMC8781769; doi:10.3390/ma15020485)
Supplement: Supplementary file 1 [file materials-15-00485-s001.zip › materials-1530848-supplementary.pdf]

materials

Supplementary materials

Nucleation of Surfactant–Alkane Mixed Solid Monolayer and Bilayer Domains at the Air–Water Interface

Hiroki Matsubara [1,2,*], Rikako Mori [1] and Eisuke Ohtomi [1]

[1] Department of Chemistry, Faculty of Science, Kyushu University, Motooka 744, Nishi-ku, Fukuoka 819-0395, Japan; cosmos.r.mori@gmail.com (R.M.); Eisuke.Ohtomi@wdc.com (E.O.)
[2] Graduate School of Advanced Science and Engineering, Hiroshima University, Kagamiyama 1-3-1, Higashi-Hiroshima 739-8526, Japan
* Correspondence: hmtbr@hiroshima-u.ac.jp

Citation: Matsubara, H.; Mori, R.; Ohtomi, E. Nucleation of Surfactant–Alkane Mixed Solid Monolayer and Bilayer Domains at the Air–Water Interface. *Materials* **2022**, *15*, 485. https://doi.org/10.3390/ma15020485

Academic Editor: Ornella Cavalleri

Received: 14 December 2021
Accepted: 6 January 2022
Published: 9 January 2022

Publisher's Note: MDPI stays neutral with regard to jurisdictional claims in published maps and institutional affiliations.

Copyright: © 2022 by the authors. Licensee MDPI, Basel, Switzerland. This article is an open access article distributed under the terms and conditions of the Creative Commons Attribution (CC BY) license (https://creativecommons.org/licenses/by/4.0/).

1. SURFACE TENSION AND SURFACE DENSITY OF SURFACTANT-ALKANE MIXED ADSORBED FILMS

Figure S1 shows the surface tension and surface density determined for the C16TAB-C14 system and Figure S2 shows those determined for the C16TAB-C16 system. The surface tension and surface density data for the C14TAB-C14 system were previously published in the reference [2]. Since the phase boundary between the L and S1 phases dependent on temperature (see Figure 2), the surface tension measurements were performed at the temperatures where the mixed adsorbed films were in the L or the S1 phase on the entire concertation range. As demonstrated in the reference [2], the surface tension - temperature curves measured at different concentrations were almost parallel and therefore the surface tension – concentration curves replotted for given temperatures were also parallel. Hence, the surface density only slightly dependent on the temperature both in the L and S1 phases. According to such a observation, we used the surface densities values obtained at temperatures designated in Figure S1 and S2 to consider the morphology of domains at the surface transition temperatures.

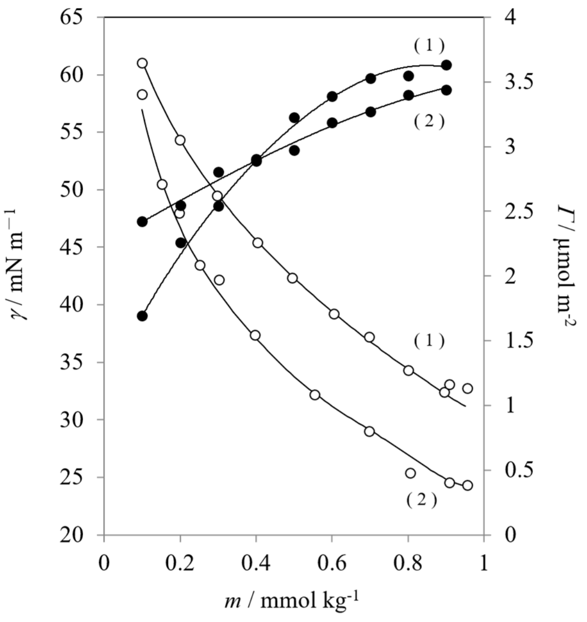

Figure S1. Surface tension (open circle) and surface density (filled circle) vs. C16TAB concentration curves in the presence of C14 at (1) 25.0 °C and (2) 10.0 °C.

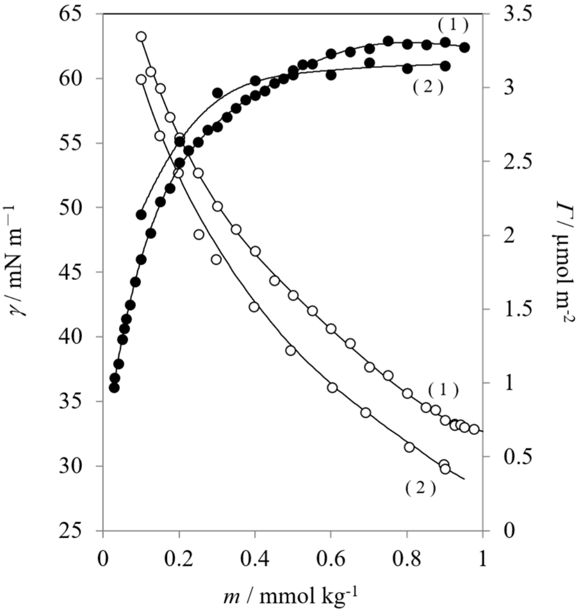

Figure S2. Surface tension (open circle) and surface density (filled circle) vs. C16TAB concentration curves in the presence of C16 at (1) 25.0 °C and (2) 19.0 °C.
